# Altered carbon status in *Glycine max* hairy roots induced by *Agrobacterium rhizogenes*

**DOI:** 10.1080/15592324.2022.2097469

**Published:** 2022-07-12

**Authors:** Satoru Okamoto, Yukiko Ueki

**Affiliations:** a Graduate School of Science and Technology, Niigata University, Niigata, Japan; b PRESTO, Japan Science and Technology Agency, Kawaguchi, Japan

**Keywords:** Starch, glucose, sucrose, *Glycine max*, *Agrobacterium rhizogenes*, hairy roots

## Abstract

Plants fix CO_2_ into carbohydrates through photosynthesis, and various organisms interact with plants to obtain carbohydrates. *Agrobacterium rhizogenes* is a soil bacterium known as a plant pathogen that induces hairy root disease. Through *A. rhizogenes*-plant interactions, transfer-DNA (T-DNA) of the Ri plasmid is inserted into the host plant genome, leading to excessive formation of hairy roots and the synthesis of opines that are carbon and nitrogen sources for *A. rhizogenes*. In this study, we analyzed the carbohydrate contents in soybean (*Glycine max*) hairy roots. We found that the starch content was strongly increased in hairy roots, whereas the glucose was significantly decreased. On the other hand, no significant differences were observed in sucrose levels between the main roots and hairy roots of *A. rhizogenes*-inoculated plants. This result suggests that *A. rhizogenes* infection caused a change in primary carbon metabolism in the host plant cells.

Plants are autotrophic organisms that fix CO_2_ into carbohydrates through photosynthesis, and carbohydrates are consumed as carbon skeletons and energy sources in many biological processes. One aim of the organisms that interact with plants, such as herbivores, parasites, pathogens and symbionts, is to acquire carbon sources from plants. It has been reported that some pathogens alter carbon metabolism in host plants. Bacterial and fungal pathogens upregulate cell wall invertases in host plants.^1,2^ It is known that cell wall invertases catabolize sucrose to glucose and fructose and play important roles in supplying carbohydrates to sink organs.^3^ Therefore, those pathogens may modify infected sites that act as sink tissues.

*A. rhizogenes* is a soil bacterium and is known as a plant pathogen that induces hairy root disease. Through *A. rhizogenes*-plant interactions, the T-DNA of the Ri plasmid is inserted into the host plant genome, and a set of genes on T-DNA, such as *root oncogenic loci* (*rol*) genes, are expressed in host plant cells.^4,5^ These genes are thought to lead to a massive growth of adventitious roots called ‘hairy roots’ and opine biosynthesis. Opines are secreted to the rhizosphere, and *A. rhizogenes* uses them as carbon and nitrogen sources. Opines are synthesized from amino acids and photoassimilates (ketoacids or sugars) in host plant cells.^6^ In addition, although the functions of the oncogenes are not fully understood, it has been reported that one oncogene, *rolC*, affects carbon status in host plant cells when it is ectopically expressed in tobacco (*Nicotiana tabacum*) leaves.^7^ Therefore, there is a possibility that plant primary metabolism is affected by *A. rhizogenes* infection. To explore this possibility, we analyzed the carbohydrate contents in soybean hairy roots.

To induce hairy roots in soybean, *A. rhizogenes* is inoculated on hypocotyls by stabbing with a needle.^8^ In this study, the hypocotyls of soybean cv. Enrei were inoculated with *A. rhizogenes* strain K599 five days after germination. The bacterial paste was picked up by a needle, and then the hypocotyls were stabbed with the needle (details are described in Kereszt et al. (2007)). After inoculation, the hypocotyls were covered with soil to induce hairy roots, and the plants were grown in a growth chamber (23°C, 16 hours light/8 hours dark). During cultivation, no marked morphological differences were observed between the shoots of inoculated and control (not inoculated) plants. Because the main roots were not excised, the inoculated soybean plants developed hairy roots while retaining the original main root systems (Figure 1a,b). Adventitious roots are also often developed from the hypocotyls; thus, to confirm the successful induction of hairy roots, we introduced *A. rhizogenes* harboring the *p35S*:GFP construct^9^ into soybean (Figure 1c). At 28 days after inoculation, we sampled the main roots and hairy roots separately and measured the mRNA levels of the *orf12* (*rolC*) and *orf13* (*rolD*) genes. The results revealed the expression of the *rol* genes exclusively in the hairy root samples (Figure 1d).

**Figure 1.**
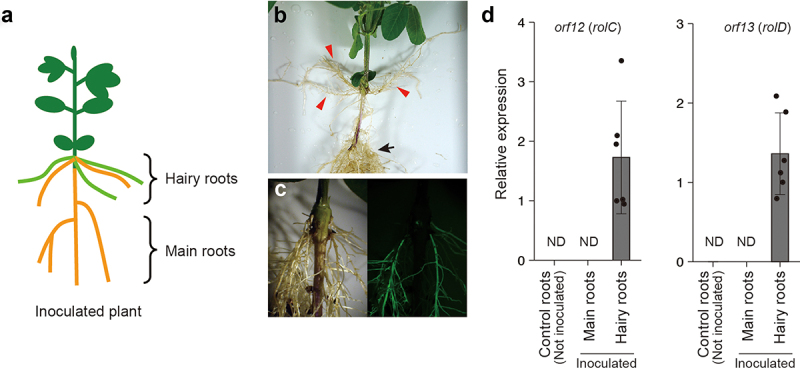
Characterization of soybean hairy roots. (a) Schematic model of soybean hairy roots. *A. rhizogenes* was infected to hypocotyls, and the hairy roots developed from the infected site. (b) Hairy roots or adventitious roots are developed from soybean hypocotyls. Red triangles indicate hairy roots or adventitious roots, and the black arrow indicates the original main root system. (c) Hairy roots are distinguishable by using a GFP marker. (d) Relative mRNA level of the *rol* genes. The expression levels of each *rol* gene were normalized to those in hairy roots. The dots represent individual measurements. Each result is the mean ± standard error of the mean of measurements obtained from four (control roots) or six (main roots and hairy roots) individual samples from two independent experiments. ND means not detected. The primers used for real-time PCR are listed in the Supplemental Table, and the conditions for real-time PCR are described in Okamoto et al. (2022).

We analyzed the carbohydrate (sucrose, glucose and starch) contents of the roots as described in Okamoto et al. (2022)^10^ (Figure 2). Sucrose is the most commonly translocated form of sugar from source leaves to sink organs. The sucrose status in the roots of *A. rhizogenes*-inoculated plants was lower than that of control plants, whereas in inoculated plants, no significant differences in sucrose levels between the main roots and hairy roots were observed. Glucose in roots is mainly derived from the photoassimilates that are translocated from source leaves and is consumed through respiration associated with growth and ion uptake and secretion to the rhizosphere as organic compounds. Interestingly, the glucose level in hairy roots was significantly lower than that in main roots or control roots. Furthermore, the content of starch, which is a storage form of carbohydrates, was remarkably higher in hairy roots than in main roots and control roots. These results suggest that primary carbon metabolism in host plant cells is affected by *A. rhizogenes* infection.

**Figure 2.**
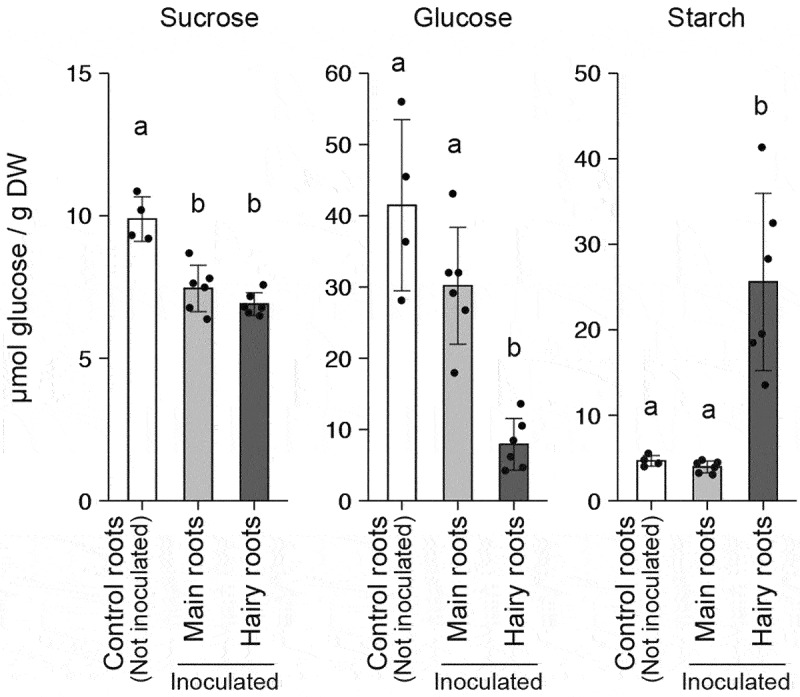
Carbohydrate levels in soybean roots. The sucrose, glucose, and starch contents are shown. The roots were sampled at the end of the day. The dots represent individual measurements. Each result is the mean ± standard error of the mean of measurements obtained from four (control roots) or six (main roots and hairy roots) individual samples from two independent experiments. Statistical differences were evaluated by one-way ANOVA (*P* < 0.001) followed by Tukey’s test (*P* < 0.001).

The hairy root transformation technique is a powerful tool to analyze the function of genes. Recently, we found peptide genes that affect carbohydrate levels in roots.^10^ However, the hairy root transformation technique may not be suitable to study such genes because carbohydrate status is affected in the hairy roots of the control line (empty vector). Other techniques, such as stable transformation experiments, are required to explore the function of genes that are supposed to be involved in carbon metabolism or partitioning.

In roots, glucose is mainly derived from the degradation of photoassimilates translocated from the source leaves and is consumed through respiration and secretion to the rhizosphere.^11^ According to Jones et al. (2009),^12^ approximately 17% of the net fixed carbon is returned to the atmosphere by rhizosphere respiration or recovered in soil residues. Some of this carbon contributes to the establishment of interactions between various soil microbes and plants. On the other hand, starch is a storage form of carbohydrates, and if necessary, the degradation of starch can supply a carbon source. In this study, we found that starch content was strongly increased in hairy roots, whereas glucose level was significantly decreased (Figure 2). This implies that *A. rhizogenes* alters carbon metabolism in host plant cells to inhibit the secretion of carbon sources, with the possible exception of opine, to the rhizosphere and store carbon sources for a continuous supply of opine. Considering that ectopic expression of one of the oncogenes, *rolC*, induces chlorosis and starch accumulation in tobacco leaves,^7^ there is a possibility that bacterial oncogenes affect carbon metabolism in hairy roots.

Sweet potato (*Ipomoea batatas*) is an important crop, and its storage roots contain large amounts of starch. It has been reported that the T-DNA sequence of *Agrobacterium* spp. is detected in the genome of cultivated sweet potato, and the inserted T-DNA fragment retains some functional open reading frames of oncogenes.^13^ Although the inserted T-DNA sequences were not identical between sweet potato and soybean in this study, considering our results, transferring the DNA fragment of *Agrobacterium* might have a positive effect on starch accumulation in storage roots and contribute to the breeding of sweet potato.

## Supplementary Material

Supplemental Material
